# Predicting long-term outcomes for acute ischemic stroke using multi-model MRI radiomics and clinical variables

**DOI:** 10.3389/fmed.2024.1328073

**Published:** 2024-03-01

**Authors:** Lai Wei, Xianpan Pan, Wei Deng, Lei Chen, Qian Xi, Ming Liu, Huali Xu, Jing Liu, Peijun Wang

**Affiliations:** ^1^Department of Medical Imaging, Tongji Hospital, School of Medicine, Tongji University, Shanghai, China; ^2^Institute of Medical Imaging Artificial Intelligence, Tongji University School of Medicine, Shanghai, China; ^3^Department of Research United Imaging Intelligence Co., Ltd., Shanghai, China; ^4^Department of Radiology, Shanghai East Hospital, Tongji University School of Medicine, Shanghai, China; ^5^Department of Radiology, Xinhua Hospital Affiliated to Shanghai Jiaotong University School of Medicine, Shanghai, China; ^6^Department of Radiology, Putuo Hospital, Shanghai University of Traditional Chinese Medicine, Shanghai, China; ^7^Department of Radiology, Zhabei Central Hospital, Shanghai, China

**Keywords:** acute ischemic stroke (AIS), prognosis, radiomics, diffusion-weighted imaging (DWI), apparent diffusion coefficient (ADC), machine learning (ML)

## Abstract

**Purpose:**

The objective of this study was to create and validate a novel prediction model that incorporated both multi-modal radiomics features and multi-clinical features, with the aim of accurately identifying acute ischemic stroke (AIS) patients who faced a higher risk of poor outcomes.

**Methods:**

A cohort of 461 patients diagnosed with AIS from four centers was divided into a training cohort and a validation cohort. Radiomics features were extracted and selected from diffusion-weighted imaging (DWI) and apparent diffusion coefficient (ADC) images to create a radiomic signature. Prediction models were developed using multi-clinical and selected radiomics features from DWI and ADC.

**Results:**

A total of 49 radiomics features were selected from DWI and ADC images by the least absolute shrinkage and selection operator (LASSO). Additionally, 20 variables were collected as multi-clinical features. In terms of predicting poor outcomes in validation set, the area under the curve (AUC) was 0.727 for the DWI radiomics model, 0.821 for the ADC radiomics model, 0.825 for the DWI + ADC radiomics model, and 0.808 for the multi-clinical model. Furthermore, a prediction model was built using all selected features, the AUC for predicting poor outcomes increased to 0.86.

**Conclusion:**

Radiomics features extracted from DWI and ADC images can serve as valuable biomarkers for predicting poor clinical outcomes in patients with AIS. Furthermore, when these radiomics features were combined with multi-clinical features, the predictive performance was enhanced. The prediction model has the potential to provide guidance for tailoring rehabilitation therapies based on individual patient risks for poor outcomes.

## 1 Introduction

Acute ischemic stroke (AIS) is a globally prevalent condition that ranks among the leading causes of disability and mortality, accounting for a staggering 60–80% of all stroke incidents (1, 2). The middle cerebral artery (MCA) territory is the most common site for AIS (3, 4). The outcomes of AIS are influenced by various factors related to patient differences, such as demographics, general health conditions, and the extent of cerebral infarction (5, 6). Predicting the prognosis of AIS quickly and accurately is essential for determining appropriate clinical management strategies (7, 8).

Radiomics (RA) is a discipline that extracts quantitative and high-dimensional features from medical images (9, 10). These features are indistinguishable to the naked eyes, but they may contain information related to the pathophysiology of diseases (11, 12). Currently, the role of RA was explored in the prediction of early outcome and long-term prognosis of AIS (13–17). However, most studies primarily predicted the outcomes of AIS based on a limited sample size of patients from either a single hospital or two hospitals (13–17). Additionally, the majority of these studies have focused on Computed Tomography (CT) images or a single-modality Magnetic Resonance Imaging (MRI) images (13, 14). Very few studies have investigated long-term outcomes for AIS using multi-modalities MRI images (15, 17), and only a few studies have developed combination models that integrated clinical and radiomic features with standard validation process (15, 16).

In this retrospective multicenter study, we developed a prediction model for long-term outcome of AIS in middle cerebral artery territory (MCA-AIS) using a combination of multi-model MRI images and multiple clinical variables from four medical centers. We extracted and selected 49 radiomics features from Diffusion-weighted imaging (DWI) and Apparent diffusion coefficient (ADC) images, and incorporated various clinical variables, such as general information, medical history, neurological scores, neuroimaging score, and laboratory examinations. Using machine learning (ML) techniques, we established models to rapidly and accurately predict the long-term outcomes of AIS. To ensure the robustness of our model, we furtherly validated it using comprehensive evaluations.

## 2 Methods

### 2.1 Study population

**Figure 1:**
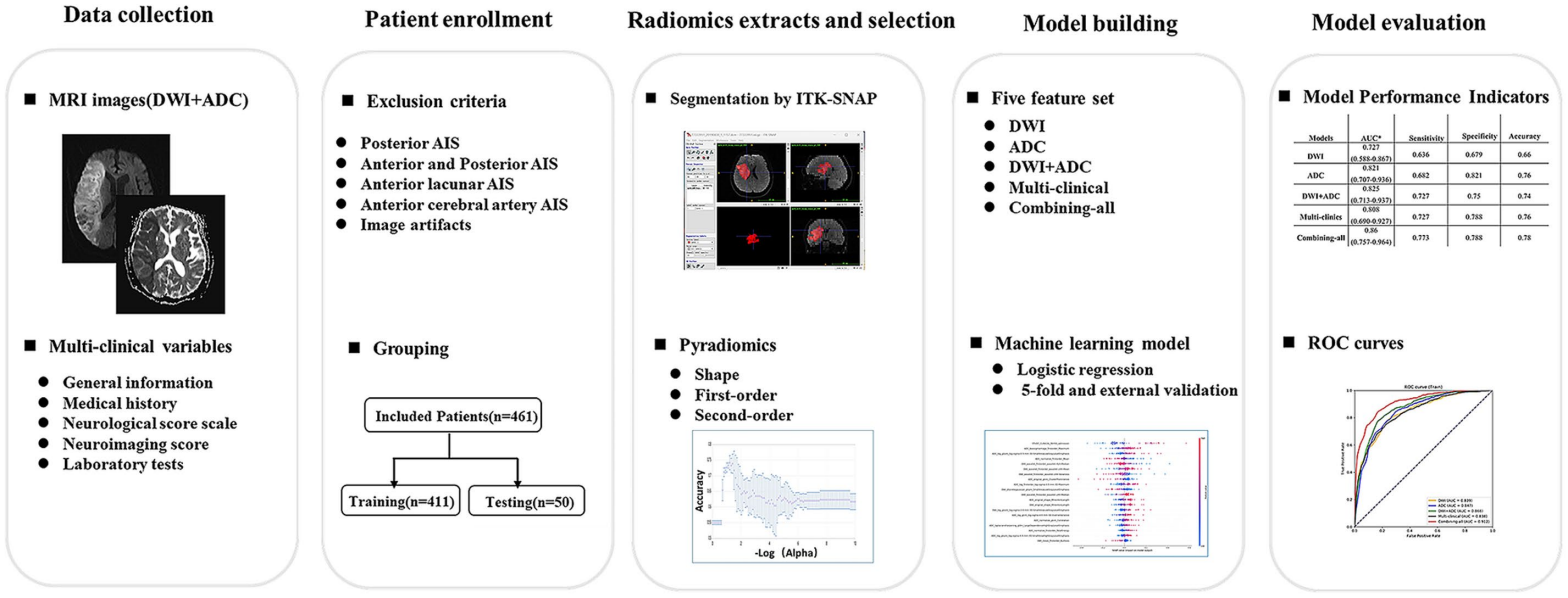
Flowchart of study patients and process. DWI = diffusion-weighted imaging, AIS = acute ischemic stroke.

This study was a retrospective multi-center investigation. The study received approval from the Ethics Review Committee of Tongji Hospital in Shanghai (Approval No. K-2020 021), written informed consent for participation was not required for this study in accordance with national legislation and the institutional requirements. We pooled individual patient-level data from patients with AIS admitted to Tongji Hospital affiliated to Tongji University, Xinhua Hospital affiliated to the School of Medicine of Shanghai Jiaotong University, East Hospital affiliated to Tongji University and Putuo Hospital affiliated to Shanghai University of Traditional Chinese Medicine from January 2018 to December 2021. The admission criteria are as follows: (1) patients who had brain MRI (including DWI and ADC images) examination within 3 days after symptom onset; (2) initially diagnosed MCA-AIS patients who were admitted to the hospital for treatment; (3) patients who underwent DWI imaging for depicting lesions with a maximum diameter more than 1.5 cm; (4) initially diagnosed MCA-AIS patients who were admitted to the hospital for standard stroke treatment. The exclusion criteria were as follows: (1) patients with AIS involving posterior circulation area; (2) patients with AIS involving the anterior cerebral artery region; (3) patients with lacunar infarcts; (4) patients with poor quality images. A total of 1,675 AIS patients were included, and 1,316 patients were excluded due to posterior cerebral AIS (*n* = 293), anterior and posterior AIS (*n* = 112), anterior lacunar AIS (*n* = 516), anterior cerebral artery cerebral AIS (*n* = 262), and image artifacts (*n* = 31). Finally, 461 cases met the inclusion criteria. All included patients were randomly divided into training cohort (411) and validation cohort (50). A flowchart of the patient selection and study process was provided in Figure 1.

### 2.2 Data collection

#### 2.2.1 Multi-model MRI images

The MRI-DWI images were obtained using four different MRI scanners. The acquisition parameters were as follows: (1) Philips Ingenia 3.0 T: TR = 2,584 ms, TE = 96.7 ms, slice thickness 6 mm, slice spacing 7 mm, field of view 23 cm × 23 cm, matrix 256 × 256, excitation times 2, echo gap 0.75 ms, b value 1,000 s/mm^2^; (2) Siemens Verio 3.0 T: TR = 4,600 ms, TE = 89 ms, slice thickness 5 mm, scanning without spacing, field of view 24 cm × 24 cm, matrix 256 × 256, echo gap 0.75 ms, b value 1,000 s/mm^2^; (3) uMR 1.5 T: TR = 5,400 ms, TE = 94 ms, slice thickness 5 mm, layer spacing 6 mm, field of view 23 cm × 23 cm, echo gap 0.75 ms, b value 1,000 s/mm^2^; (4) GE SIGNA EXCITE 1.5 T: TR = 6,000 ms, TE = 81.1 ms, slice thickness 7 mm, slice spacing 8 mm, field of view 23 cm × 23 cm, matrix 256 × 256, excitation times 2, echo gap 0.75 ms, b value 1,200 s/mm^2^. The ADC images were automatically created from DWI scans using built-in software.

#### 2.2.2 Multi-clinical variables

The following 20 clinical data were collected: (1) General information: gender and age; (2) Medical history: history of smoking, history of alcohol, history of diabetes, history of myocardial infarction, history of coronary atherosclerosis, history of atrial fibrillation, history of hypertension and history of stroke; (3) Neurological score scale: National Institutes of Health Stroke Scale (NIHSS) on admission; (4) Neuroimaging score: diffusion weighted imaging-Alberta Stroke Program Early CT Score (DWI-ASPECTS); (5) Laboratory tests on admission: prothrombin time (PT), fibrinogen, D-dimer, serum Troponin I, blood glucose, blood lipids, and plasma brain natriuretic peptide (BNP).

### 2.3 Image preprocessing and delineation

Three attending neuro-radiologists manually delineated the ischemic lesions on MRI-DWI images using ITK-SNAP software (Version 3.8.0, available at http://www.itksnap.org). The ischemic lesion volume of interest (VOI) was also replicated from the DWI images onto another parametric map (ADC) and further refined by the radiologists. Finally, all the delineations were reviewed by two chief radiologists with 8 years of experience in brain imaging. All parametric maps underwent a normalization using maximum and minimum truncation processing.

### 2.4 Radiomics extraction and selection

The flowchart of radiomics analysis was shown in Figure 1. 14 image filters (such as BoxMean, AdditiveGaussianNoise, BinomialBlurImage, CurvatureFlow, BoxsigmaImage, LoG with sigma values of 0.5, 1, 1.5, and 2), Wavelet filters (LLL, LLH, LHL, LHH, HLL, HLH, HHL, HHH), Normalize, LaplacianSharpening, DiscreteGaussian, Mean, SpeckleNoise RecursiveGaussian and ShotNoise were used to generate derived images. From these derived images, first-order statistics and texture features were extracted. A total of 2,264 radiomics features were automatically extracted from each ischemic lesion. These features can be categorized into three groups: 14 shape features, 450 first-order features that quantify the distribution of voxel intensities in the images, and 1800 texture features. The texture features consist of 525 gray level co-occurrence matrix (GLCM) features, 350 gray level run length matrix (GLRLM) features, 400 gray level size zone matrix (GLSZM) features, 400 neighboring gray tone difference matrix (NGTDM) features, and 125 gray level dependent matrix (GLDM) features. These texture features capture regional heterogeneity differences. All radiomics features were normalized using Z-score.

We employed LASSO selection to identify the most reliable predictive radiomic features. Initially, we performed feature selection separately for each sequence of DWI and ADC modalities. Then, an additional round of LASSO selection was conducted to combine the selected features from both modalities, resulting in a set of multi-modality RA features. These multi-modality RA features, along with clinical features, were subsequently merged and subjected to another round of LASSO selection to obtain a comprehensive combined feature set.

Based on Harrell’s guideline, the number of selected features should be less than 10% of the sample size. Consequently, in our experiment involving the DWI sequence, ADC sequence, multi-modality sequence, and the final combination of radiomics with clinical features, the final number of selected features was approximately 30.

### 2.5 Prediction model

#### 2.5.1 Predictive task

The objective of our predictive task was to accurately predict the long-term prognosis of initially diagnosed MCA-AIS patients. The long-term prognosis was defined based on a 90-day modified Rankin Scale (90d-mRS) score, where scores of 0–2 indicated a good outcome and scores of 3–6 indicated a poor outcome. The majority of the 90-d mRS data were collected through telephone interviews, outpatient care, and clinical medical records. During phone interviews, patients were asked about their functional recovery 90 days after therapy.

#### 2.5.2 Development and validation of the predictive model

Based on multi-model MRI RA features and/or multi-clinical features, three machine learning models were constructed for binary classification (good outcome or poor outcome) by using three classifiers, namely random forest (RF), support vector machine (SVM), and logistic regression (LR). The prediction model utilized input data from one of five feature sets: (1) DWI RA features with 25 variables, (2) ADC RA features with 24 variables, (3) DWI + ADC RA features with 35 variables, (4) Multi-clinical features with 12 variables, and (5) Combining-all features with 30 variables. To optimize performance, a grid search was conducted on different features and classification algorithms for parameter tuning.

### 2.6 Statistical analysis

Mann–Whitney U test and chi-square test were used for evaluating significant differences in the variables (such as age, NIHSS score) between the training set and the validation set. The receiver operating characteristic curve (ROC) was drawn, and various performance metrics including sensitivity (SEN), specificity (SPE), accuracy (ACC), F1-Score, and area under the curve (AUC) were calculated to assess the model’s performance. The Shapley additive explanation (SHAP) diagram was utilized for model explanation. A two-tailed statistical test was used and *p*-value lower than 0.05 was considered to be statistically significant. The R software package (version 4.0.3) was used to process the demographic data for evaluating significant differences in the variables between the training set and the validation set. Python (version 3.6) was used for programming the training, validation of the prediction model, as well as conducting statistical analysis.

## 3 Results

### 3.1 Basic characteristics

As shown in Table 1, the basic variables of most of the patients showed no statistical differences (*p* > 0.05) between the training set and the validation set, such as general conditions (gender and age), medical history (hypertension, diabetes), neurological score scales (NIHSS), and laboratory tests (BNP, etc.).

**Table 1:** Basic patient information.

	Training set (*n* = 411)	Validation set (*n* = 50)	*p*-values
Basic characteristics			
Age (Median, IQR)	71 (63, 82)	65 (55.25, 83.75)	0.194
Male (Percentile: %)	259 (56.2%)	25 (5.4%)	0.074
Neurological score scale (Median, IQR)			
NIHSS on admission	5 (5, 10)	5 (3.25, 8.75)	0.253
Location (Left: Percentile: %)	228 (49.5%)	26 (5.6%)	0.641
Neuroimaging score scale (Median, IQR)			
DWI-ASPECTS	8 (6, 9)	8 (6, 8.75)	0.752
History (Percentile: %)			
Alcohol	106 (23%)	11 (2.4%)	0.561
Smoking	167 (36.2%)	17 (3.7%)	0.366
Myocardial infarction	405 (87.9%)	49 (10.6%)	0.555
Coronary atherosclerosis	86 (18.7%)	7 (1.5%)	0.249
Atrial fibrillation	74 (16.1%)	10 (2.2%)	0.730
Hypertension	295 (64%)	32 (6.9%)	0.253
Stroke	103 (22.3%)	14 (3%)	0.652
Diabetes	135 (29.3%)	14 (3%)	0.489
Laboratory test (Median, [IQR])			
Prothrombin time	11.5 (10.9, 12.3)	11.25 (10.8, 11.675)	0.023
Fibrinogen	2.97 (2.55, 3.78)	2.8 (2.405, 3.475)	0.072
D-dimer	0.56 (0.27, 1.355)	0.54 (0.25, 1.145)	0.526
Serum troponin I	0.01 (0.01, 0.0305)	0.01 (0.01, 0.019)	0.048
Blood sugar	6.46 (5.605, 8.595)	6.185 (5.235, 7.145)	0.035
Blood lipids	1.21 (0.96, 1.61)	1.21 (1.04, 1.5775)	0.839
Brain natriuretic peptide	103.1 (64.15, 269.3)	103.1 (58.275, 154.95)	0.395
Long-term outcome			
Poor outcome (90d-mRS>2) (Percentile: %)	184 (39.9%)	22 (4.8%)	0.918

IQR: interquartile range; NIHSS: National Institute of Health stroke scale; 90d-mRS:90 days-modified Rankin scale.

### 3.2 Assessment of radiomic features

**Figure 2:**
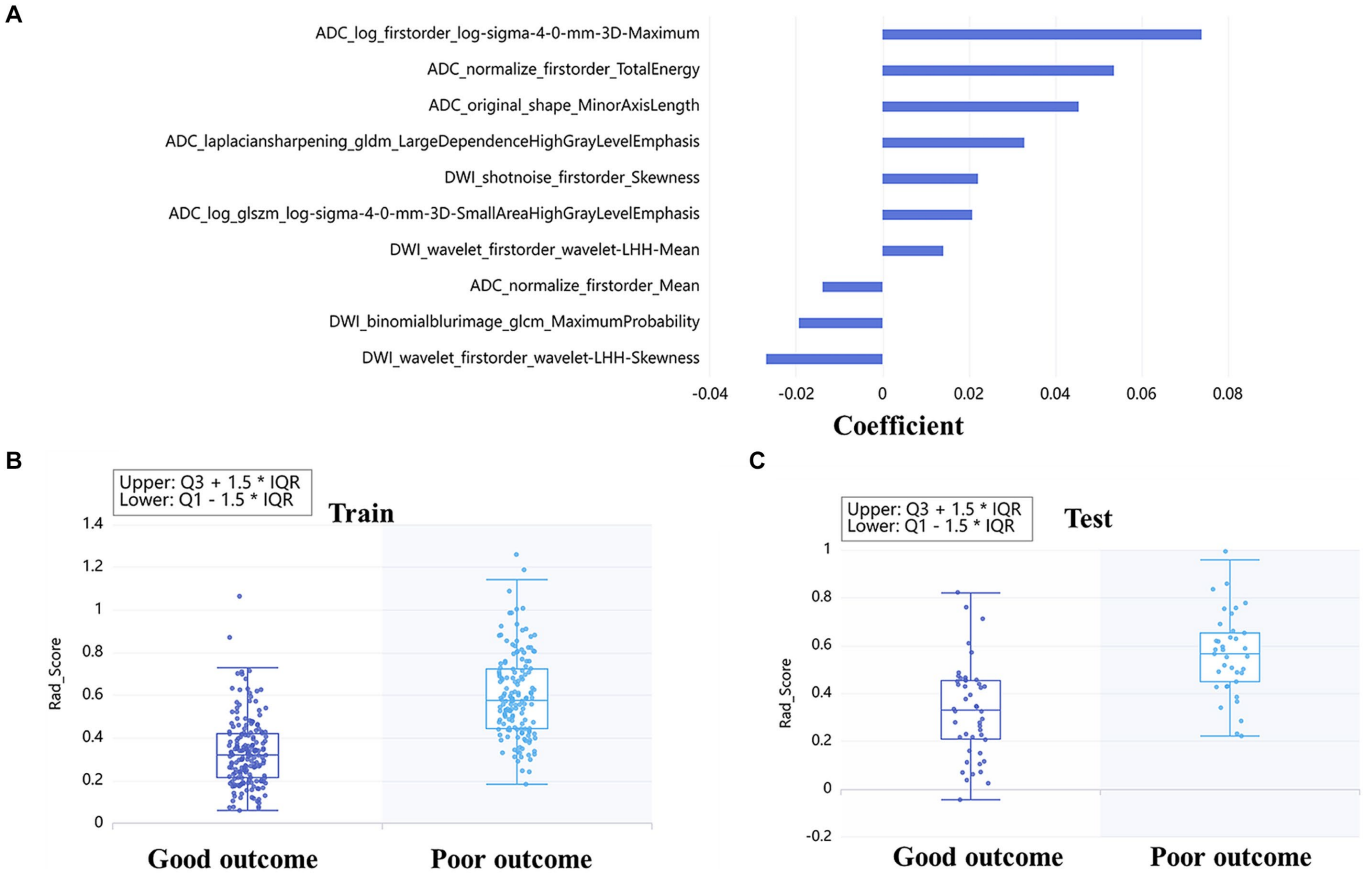
Radiomics based on DWI + ADC feature set for long-term prognosis prediction: the coefficient of each feature was taken to make a bar chart, which showed the importance of the top 10 feature in the prediction **(A)**. The box diagrams of rad-score for discriminating good and poor outcomes in the train **(B)** and test sets **(C)**.

A total of 4,528 radiomics features were extracted from DWI and ADC images. The optimal feature subset for the machine learning models consisted of 49 radiomic features, with 25 features selected from DWI and 24 features selected from ADC. These features were comprised of 4 shape features, 16 first-order features, and 29 texture features. The detailed information about the features based on DWI + ADC model was presented in Figure 2A. Rad-score was calculated according to the coefficient of the selected features, and the distribution of rad-score between good and poor outcome was shown both in train (Figure 2B) and test (Figure 2C) sets.

### 3.3 Comparison of prediction models

#### 3.3.1 Comparison between different models

**Figure 3:**
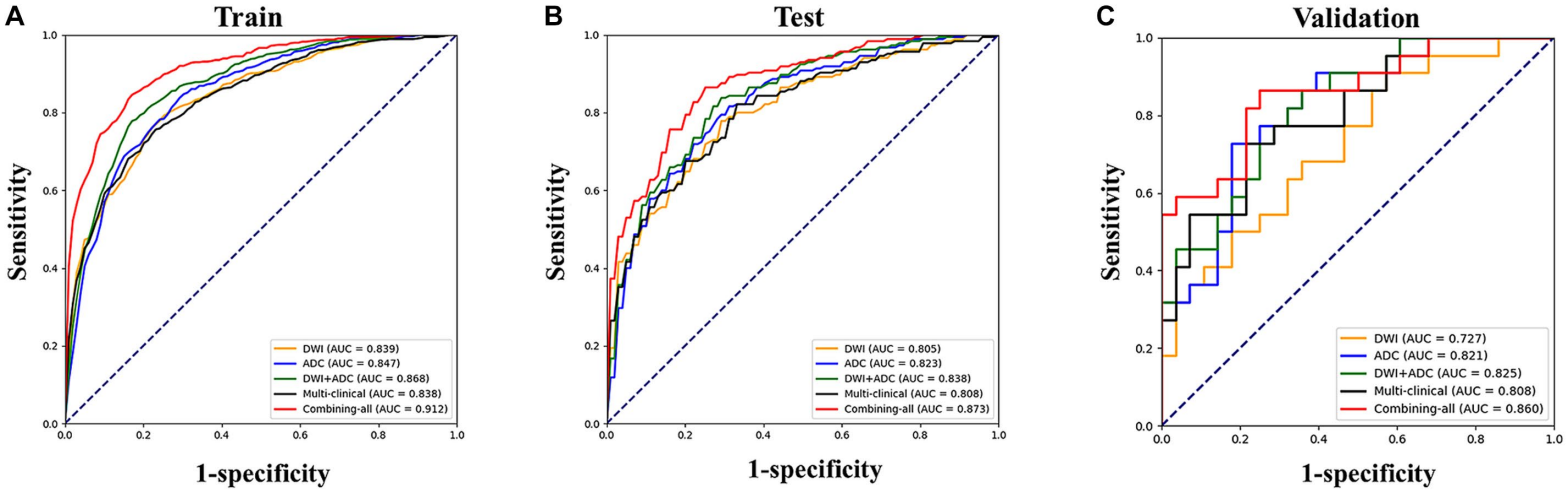
Performances of machine learning models for prediction of outcome: Receiver operating characteristic (ROC) curves of five feature sets when using the logistic regression classifier in the train **(A)**, test **(B)** and validation **(C)** set, respectively.

The LR model achieved the best classification results in all feature sets in our study. Table 2 and Figure 3 illustrated the AUC values along with other diagnostic performance metrics such as specificity, sensitivity, accuracy, and F1 Score, which demonstrated the indicator results for predicting poor outcomes in the training set, test set, and validation set. In the test set for predicting poor outcome using LR model, the AUCs were as follows: DWI RA model 0.805, ADC RA model 0.823, DWI + ADC RA model 0.838, multi-clinical model 0.808. When combining multi-clinical features and RA features, the AUC was significantly increased, reaching to 0.873. In the validation set for predicting poor outcome, the AUCs were as follows: DWI RA model 0.727, ADC RA model 0.821, ADC + DWI RA model 0.825, multi-clinical model 0.808. When combining all the features, the AUC value was increased to 0.86, which means the model with combining-all features achieved superior diagnostic performance compared to other models.

**Table 2:** The performance of the prediction models.

	Models	AUC^*^		Sensitivity		Specificity		Accuracy		F1 score	
Training (*n* = 411)		Train	Test	Train	Test	Train	Test	Train	Test	Train	Test
	DWI	0.839 (0.797–0.882)	0.805 (0.711–0.903)	0.776	0.73	0.763	0.748	0.769	0.74	0.751	0.71
	ADC	0.847 (0.806–0.889)	0.823 (0.732–0.915)	0.791	0.762	0.746	0.734	0.766	0.747	0.752	0.728
	DWI + ADC	0.868 (0.83–0.907)	0.838 (0.753–0.925)	0.85	0.838	0.738	0.721	0.788	0.774	0.783	0.768
	Multi-clinics	0.838 (0.796–0.882)	0.808 (0.714–0.903)	0.759	0.724	0.764	0.752	0.762	0.74	0.742	0.712
	Combining-all	0.912 (0.883–0.944)	0.873 (0.802–0.949)	0.847	0.805	0.815	0.801	0.83	0.803	0.817	0.783
Validation (*n* = 50)											
	DWI	0.727 (0.588–0.867)		0.636		0.679		0.66		0.622	
	ADC	0.821 (0.707–0.936)		0.682		0.821		0.76		0.714	
	DWI + ADC	0.825 (0.713–0.937)		0.727		0.75		0.74		0.711	
	Multi-clinics	0.808 (0.690–0.927)		0.727		0.788		0.76		0.727	
	Combining-all	0.86 (0.757–0.964)		0.773		0.788		0.78		0.756	

^*^AUC = area under the receiver operating characteristic curve; DWI = diffusion-weighted imaging; ADC = apparent diffusion coefficient.

#### 3.3.2 Model interpretability

**Figure 4:**
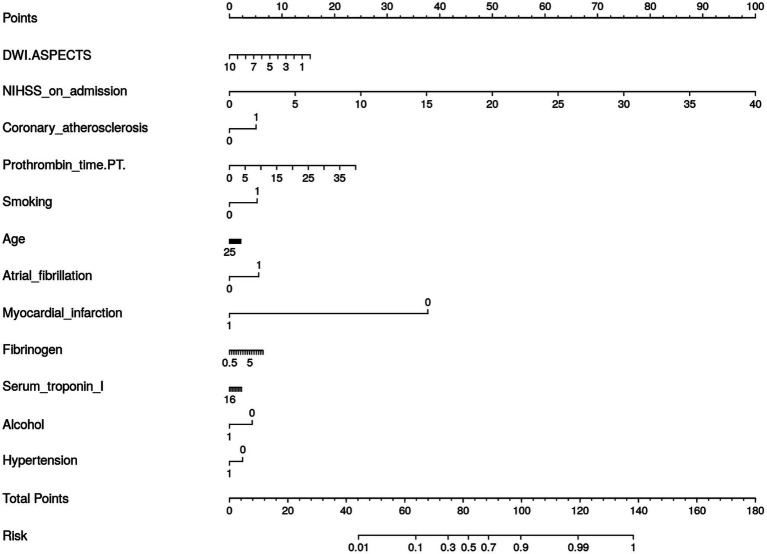
Nomogram for the Multi-Clinical model for long-term prognosis prediction.

We generated a nomogram to predict the probability of long-term outcomes using the multi-clinical feature set (Figure 4). It showed that patients with higher NIHSS on admission, a history of myocardial infarction, and lower DWI-ASPECTS were at greater risks for poor outcome.

**Figure 5:**
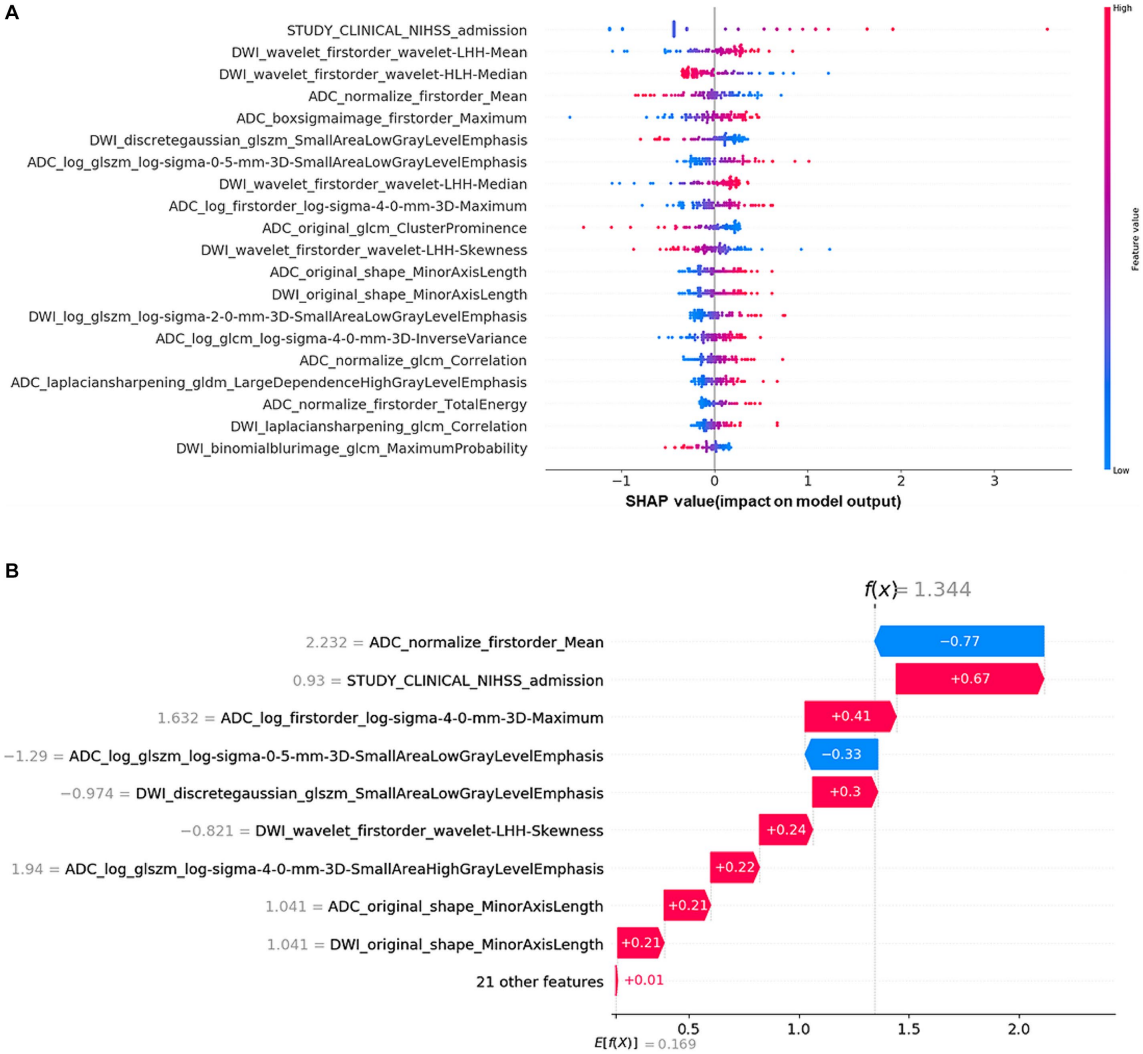
Shapley additive explanation (SHAP) diagram of variable contributions for the optimal (combining-all) predictive model: **(A)** The relative contributions of RA features and multi-clinical variables for long-term prognosis prediction. The color intensity of the graph revealed a discernible pattern: an increment in the NIHSS admission score corresponded to an escalating probability of a poor outcome. For the DWI_Wavelet_firstorder_wavelet-lhh-mean feature, the lower the value of the feature, the higher likelihood of a poor outcome. **(B)** SHAP values of a typical patient from the positive group (poor outcome), illustrated with the important variables.

The Shap values corresponding to each feature in combining-all model were also calculated. In each prediction, a positive Shap value denoted an elevated risk of poor outcome, while a negative value suggested the opposite. The accompanying Figure 5A presented the average Shap values for each feature within the test set. Notably, the NIHSS admission emerged as the most influential predictor in forecasting long-term outcomes. Alongside, several RA features, such as ADC_log_boxsigmaimage_firstorder_maximum, ADC_ normalize_firstorder_mean, and DWI_Wavelet_firstorder_wavelet-lhh-mean also played an important role in this predictive model. The detailed SHAP values of the most important variables for one typical patient from the validation group (poor outcome) was illustrated in Figure 5B.

## 4 Discussion

In this retrospective multicenter study, we developed a logistic regression model based on DWI RA features, ADC RA features, and multi-clinical factors to predict long-term outcomes in patients with AIS. Our model was applicable to MCA-AIS patients receiving different therapies and provided preferable accuracy. It was worth mentioning that our study just conformed to the “big data” trend of medicine which took radiomics as a block of “big data”.

RA can provide quantitative morphological and texture features based on voxel level while our naked eye can only distinguish 16 gray scales (18). In this context, regarding the heterogeneity of AIS lesions, radiomics seems to be superior to conventional imaging visual analysis (19, 20). As we know, ADC images can more accurately reflect diffusion restriction than DWI images without the influence of T2 shining-through effect. In this study, we found that the prediction model with ADC RA features performed better than the model with DWI RA features that was consistent with the principle of diffusion sequence imaging mentioned above, and a previous study has also yielded similar results (21). First-order features (ADC_boxsigmaimage_firstorder_Maximum) was positively correlated to infarction core volume, which was considered to be critical factors for stroke severity and treatment plan in the guidelines. And Vogt et al. have reported that the initial lesion volume of cerebral infarction acted as an independent predictor of prognosis (90d-Rankin score) (22). Our study included MCA-AIS cases in 72 h from onset, predominantly capturing patients in the acute-subacute phase. There was vascular edema and/or cytotoxic edema in acute-subacute cerebral infarction, that was the pathophysiology mechanism of the signal elevation on DWI images and signal reduction on ADC maps. First-order feature (ADC_normalize_firstorder_Mean, DWI_wavelet_firstorder_wavelet-LHH-Mean) showed the average gray level intensity within the infarction core, and both higher gray level intensity on DWI and lower gray level intensity on ADC reflected more severe overall diffusion restriction within the lesion, suggesting a higher grade of overall edema. Consequently, we hypothesized that the voxel-based diffusion restriction heterogeneity represented the progress rate of blood–brain barrier destruction. First-order feature (ADC_normalize_firstorder_totalenergy) measured the magnitude of voxel values in images, with larger values indicating a higher sum of the squares of these values. This metric suggested that the infarction core on ADC images of patients with poor outcomes had more heterogeneity. A two-center study showed that infarction lesion homogeneity of DWI images indicated favorable outcomes, which was similar with our results (16).

This study collected multiple-dimension clinical variables, including general information, medical history, neuroimaging scores, and laboratory test, which was different from previous studies (13–17). We observed that NIHSS score on admission remained associated with the risks for poor outcomes whether in multi-clinical model or combing-all model. The National Institute of Health Stroke Scale (NIHSS) is the most commonly used clinical score (23), which quantitatively and comprehensively evaluates the functional impairment in stroke patients. A history of myocardial infarction, indicative of underlying atherosclerotic disease, and a shortened prothrombin time, suggestive of hypercoagulability, are both significant risk factors for the onset and progression of AIS (24). We also found that DWI-ASPECTS played an important role in the multi-clinical model. It is a 10-point semi-quantitative scoring system for assessing the degree of ischemic changes (25, 26). ASPECTS has been widely utilized to identify patients that presumed to have a large ischemic core and high risks for intracerebral hemorrhage and poor clinical outcome (27, 28). These findings are consistent with the current guidelines and consensus for the diagnosis and therapy of AIS (29, 30). However, medical history and laboratory test contributed little to the prediction models in our study.

Despite the favorable prognostic efficacy of the combining model, our research still has some limitations. First, a more extensive and prospective study cohort is needed to generalize the performance of the prediction model in the future. However, compared with most previous studies (13–17), our sample size had certain advantages, especially the four-center characteristic. Second, reperfusion factors, such as collateral circulation and vascular recanalization, have not been investigated as a variable. Third, when collecting the data, the lacunar cerebral infarction patients with good prognosis were excluded, which meant that this study did not include all clinically common cases of AIS.

## 5 Conclusion

Our findings highlighted the utility of radiomics based on DWI and ADC images in predicting long-term outcomes in patients with MCA-AIS. The prediction model, which incorporated multi-clinical variables along with ADC + DWI RA features, demonstrated the highest efficiency in the prediction of long-term outcomes for AIS. This model has the potential to assist clinicians in offering personalized management strategies for optimal patient care.

## Data availability statement

The raw data supporting the conclusions of this article will be made available by the authors, without undue reservation.

## Ethics statement

The studies involving humans were approved by Ethics Committee of Shanghai Tongji Hospital. The studies were conducted in accordance with the local legislation and institutional requirements. Written informed consent for participation was not required from the participants or the participants’ legal guardians/next of kin in accordance with the national legislation and institutional requirements.

## Author contributions

LW: Conceptualization, Data curation, Formal analysis, Methodology, Writing – original draft, Investigation, Visualization. XP: Formal analysis, Methodology, Validation, Visualization, Writing – original draft. WD: Methodology, Validation, Visualization, Writing – original draft. LC: Conceptualization, Methodology, Supervision, Writing – original draft. QX: Data curation, Investigation, Writing – review & editing. ML: Data curation, Investigation, Writing – review & editing. HX: Data curation, Investigation, Writing – review & editing. JL: Conceptualization, Project administration, Supervision, Writing – review & editing. PW: Conceptualization, Funding acquisition, Resources, Supervision, Writing – review & editing.
